# Omitting ALND Is Not Safe for a Cohort of Early-Stage Breast Cancer Patients with 1–2 SLNs Macro-Metastases and Breast-Conserving Therapy: A Single-Center Retrospective Study

**DOI:** 10.18502/ijph.v49i7.3579

**Published:** 2020-07

**Authors:** Xiangyu WANG, Yinqi GAO, Xue YANG, Xiangyi KONG, Zixing WANG, Yi FANG, Jing WANG

**Affiliations:** 1. Department of Breast Surgical Oncology, National Cancer Center/National Clinical Research Center for Cancer/Cancer Hospital, Chinese Academy of Medical Sciences and Peking Union Medical College, Beijing, 100021, China; 2. Department of Oncology, Capital Medical University Electric Power Teaching Hospital, Beijing, 100073, China; 3. School of Basic Medical Sciences, Jining Medical University, Jining, 272067, China

**Keywords:** Breast cancer, Risk-factors, Predictive model, Macro-metastases, Breast-conserving surgery

## Abstract

**Background:**

Omitting axillary lymph node dissection (ALND) is recommended for early-stage breast cancer patients with 1–2 sentinel lymph nodes (SLNs) macro-metastases and breast-conserving therapy. However, it is not safe for part of patients, so it is significant to find risk factors and develop a predictive model of non-SLNs metastases in breast cancer patients with 1–2 SLNs macro-metastases and breast-conserving therapy.

**Methods:**

This retrospective study enrolled 228 breast cancer patients with 1–2 SLNs macro-metastases who underwent ALND and breast-conserving surgery between Jan 2012 and Dec 2017 at Cancer Hospital Chinese Academy of Medical Sciences. Chi-square test and backward stepwise binary logistic regression were used to find factors that influenced non-SLN metastases, then a predictive model was formulated and obtained its area under the curve.

**Results:**

Tumor pathologic invasion size, number of positive SLNs and ALN status on imaging was associated with non-SLNs metastases. The predictive model was also formulated based on these three factors to assess and the area under the curve of model was 0.708.

**Conclusion:**

We developed a predictive model to assess the high-risk cohort of patients of non-SLNs metastases which can be an auxiliary tool for doctors.

## Introduction

Breast cancer has been the most common malignant tumor for women all over the world. With the development of surgery and radiotherapy, breast conserving surgery followed radiotherapy, and sentinel lymph node biopsy (SLNB) has become standard treatments for early invasive breast cancer patients. Traditionally, patients with positive sentinel lymph nodes (SLNs) underwent axillary lymph node dissection (ALND) to assess the status of axillary lymph nodes and possible metastatic lymph nodes also could be excised. However, ALND sometimes caused some side effects such as lymphedema, nerve injury, and shoulder dysfunction, which would influence the function and quality of life. Furthermore, only ∼40% of patients with positive SLNs had metastases tumor in non-sentinel lymph nodes (non-SLNs), the other ∼60% of patients did not benefit from ALND (1–3).

Results reported by several randomized prospective trials recommended that non-SLNs metastases may be killed by systemic chemotherapy and radiotherapy (4). Furthermore, National Comprehensive Cancer Network (NCCN) Guidelines for Breast Cancer advises that patients who meet all the 5 criteria (T1 or T2 tumor, 1 or 2 positive sentinel lymph nodes, Breast-conserving surgery, Whole-breast radiotherapy planned and No pre-operative chemotherapy) need no further axillary surgery. The recommendation is mainly based on the American College of Surgery Oncology Group (ACOSOG) Z0011 trial, which included 891 patients with T1 or T2 breast cancers and 1 or 2 positive SLNs (5). These patients were randomly assigned to two groups: patients in one group received ALND and the other group received SLNB alone. Then, the Z0011 trial came to the results that comparing to the SLNB group, the ALND group had noninferiority at a median follow-up of 9.3 years. However, the Z0011 trial has been criticized for including ∼50% of patients with only micro-metastases (metastatic tumor size between 0.2mm and 2.0mm) in SLNs, which shows low tumor burden in axillary lymph nodes (ALNs) (6, 7). Another randomized trial (IBCSG 23–01) has showed that ALND could be avoided in patients, but patients included had only micro-metastases in SLNs and the rate of non-SLNs involved was only 13% (8).

Therefore, it takes us one question that omitting ALND may not be safe for some early-stage breast cancer patients with 1–2 sentinel lymph nodes macro-metastases and breast conserving therapy. The subgroup of patients who have macro-metastases and receive breast conserving surgery is a grey zone, though previous studies have investigated factors influencing non-SLNs metastases. We attempt to determine the risk factors of non-SLNs metastases and develop a model to predict non-SLNs metastases to find the subgroup of patients who may should receive ALND based on the data in a single cancer center.

## Methods

### Study population

Between Jan 2012 and Dec 2017, 228 breast cancer patients treated at Cancer Hospital Chinese Academy of Medical Sciences (Beijing, China) were included. Patients were eligible for the study if they met the following conditions: T1-T2 breast tumor, breast-conserving surgery, successful SLNB with pathologically proven 1–2 SLNs macro-metastases (at least one metastasis greater than 2.0 mm) and subsequent ALND were performed, first-time breast cancer diagnosis and no neoadjuvant systemic therapy.

This study has been approved by the Institutional Ethics Review Board of National Cancer Center/National Clinical Research Center for Cancer/Cancer Hospital, Chinese Academy of Medical Science and Peking Union Medical College.

### SLNB procedure

Sentinel nodes were located by using a combined technique of radio-colloid and blue dye injection. Two mCi of 99mTc-dextran and1 ml of methylene blue were injected in the sub-areolar 2–6 h and 5–10 min before surgery separately. All hot, blue, and palpably suspicious lymph nodes were dissected and submitted for frozen sectioning. ALND was performed in patients with positive SLNs. Nodes obtained from SLNB and ALND were submitted for routine histopathology.

### Data collection

The data collected from patients were shown in Table 1. Classifications of histological grade, ER, PR, HER2, Ki-67 were according to NCCN Guidelines Insights: Breast Cancer, Version 1.2018.

**Table 1:** Univariable associations of factors for non-SLNs metastases

***Predictors***	***Patients (n, %)***	***Non-SLNs positive patients (n, %)***	***P-value***
Age of diagnosis(yr)			0.503
≤50	125(54.8%)	42(33.6%)	
>50	103(45.2%)	39(37.9%)	
Tumor location			0.072
left	114(50.0%)	34 (29.8%)	
right	114 (50.0%)	47 (41.2%)	
Pathologic invasion size			0.009
≤1cm	23(10.1%)	4(17.4%)	
>1cm, ≤2cm	145(63.6%)	47(32.4%)	
>2cm	60(26.3%)	30(50%)	
Histological type			0.513
invasive ductal carcinoma	151(66.9%)	50(33.1%)	
invasive ductal carcinoma with carcinoma in situ	68(29.8%)	28(41.2%)	
others	9(3.9%)	3(33.3%)	
Histological grade			0.560
I	16(7.0%)	5(31.3%)	
II	156(68.4%)	59(37.8%)	
III	47(20.6%)	14(29.8%)	
unkown	9(4.0%)	3(33.3%)	
Lympho-vascular invasion			0.165
yes	66(28.9%)	28(42.4%)	
no	162(71.1%)	53(32.7%)	
Multifocality			0.926
yes	10(4.4%)	1(1.2%)	
no	218(95.6%)	80(36.7%)	
Number of identified SLNs			0.029
1–2	43(18.9%)	22(51.2%)	
3–4	120(52.6%)	42(35.0%)	
>4	65(28.5%)	17(26.2%)	
Extranodal extension			0.636
Yes	12(5.3%)	3(25.0%)	
No	216(94.7%)	78(36.1%)	
ER			0.401
positive	205(89.9%)	71(34.6%)	
negative	23(10.1%)	30(43.5%)	
PR			0.228
positive	197(86.4%)	67(34.0%)	
negative	31(13.6%)	14(45.2%)	
HER-2			0.117
positive	36(15.8%)	18(50.0%)	
negative	177(77.6%)	60(33.9%)	
unknown	15(6.6%)	3(20.0%)	
Ki-67			0.861
≤14%	55(24.1%)	19(34.5%)	
>14%	173(75.9%)	62(35.8%)	
ALN status on imaging			<0.001
normal	177(77.6%)	52(29.4%)	
abnormal	51(22.4%)	29(56.9%)	
Body mass index			0.402
<24	106(46.5%)	34(32.4%)	
≥24	122(53.5%)	46(37.7%)	

Non-SLNs=non-sentinel lymph nodes, SLNs=sentinel lymph nodes, ER=estrogen receptor, PR=progestogen receptor, HER-2=human epidermal growth factor receptor-2

### Statistical analysis

All statistical analyses were performed using SPSS version 19.0 (IBM, Armonk, NY, USA) with the significance level set at *P*<0.05. Chi-square test was used to do univariate analysis of the associations between non-SLNs metastases and clinical-pathological factors.

Then, factors of *P*<0.05 in univariate analysis were brought to do multivariate analysis by binary logistic regression.

Predictive model was formulated based on predictive factors resulted in factors with *P*-value less than 0.05 in multivariate analysis. The performance of the predictive model was assessed by the area under the receiver operating characteristic curve (ROC). Area under the curve (AUC) ranging from 0.5 to 1.0 represents the probability that a randomly selected patient with the outcome (non-SLNs metastases) has a greater risk prediction than a randomly selected patient without the outcome.

## Results

### Clinical characteristics

It consisted of 228 breast cancer patients with 1–2 SLNs metastases, breast-conserving surgery and an age range of 26–79 years. The mean number of identified SLNs was 3.8 and non-SLNs metastases were observed in 81(35.5%). Most histological type (96.1%) was invasive ductal carcinoma with/without carcinoma in situ. All descriptive characteristics of the study population are listed in Table 1.

### Risk factors and predictive model

Table 1 also shows the relationships between clinic-pathological variables and non-SLNs metastases. Univariate analysis showed non-SLNs involvement had a significant association with pathologic invasion size (*P*=0.009), number of identified SLNs (*P*=0.029), and ALN status on imaging (*P*<0.001).

As shown in Table 2, by backward stepwise binary logistic regression, pathologic invasion size (*P*=0.016), number of identified SLNs (*P*=0.012), and ALN status on imaging (*P*=0.002) were significantly associated with non-SLNs involvement. Then we developed the prediction model as follows:

$$p=\frac{\text{exp}(-1.131+0.642*\text{S}-0.548*\text{N}+1.029*\text{A})}{1+\text{exp}(-1.131+0.642*\text{S}-0.548*\text{N}+1.029*\text{A})}$$

*p* is the probability of non-SLNs metastases, S is equal to 1(≤1cm), 2(>1cm, ≤2cm) and 3(>2cm). N is equal to 1(1–2), 2(3–4) or 3(>4) for number of identified SLNs and A represents ALN status on imaging (0 normal or 1 abnormal).

**Table 2:** Predictive factors for non-SLNs metastases resulted from multivariate analysis

***Predictive factors***	***OR***	***95%CI***	***P-value***
Pathologic invasion size	1.901	1.128–3.202	0.016
Number of identified SLNs	0.578	0.376–0.888	0.012
ALN status on imaging	2.797	1.439–5.436	0.002

### Model performance

**Fig. 1:**
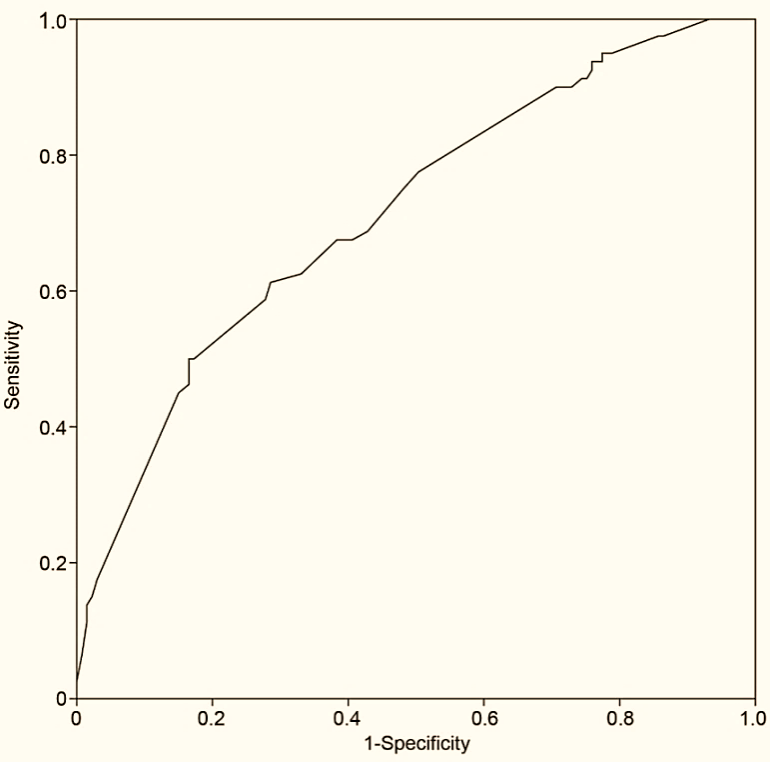
The ROC showing the performance of our model and AUC=0.708 (95%CI: 0.637–0.778)

ROC curve was shown in Fig. 1 and the AUC was 0.708 (95%CI: 0.637–0.778). A useless prediction model, such as a coin flip, would result in an AUC of 0.5, whereas the model discriminates perfectly when the AUC is 1.

## Discussion

In our study, pathologic invasion size of tumor was a predictive factor of non-SLNs metastases (*P*<0.05). With pathologic invasion size increasing, the ratio of non-SLNs metastases increased significantly. Previous investigators have also reported that larger pathologic invasion size was a significant predictive factor for non-SLNs metastases (9–18).

The present study found that number of identified SLNs was a significant factor that influenced non-SLN metastases. As shown in Table 1, more than 50% patients with only 1–2 SLNs removed had non-SLNs metastases.

With the number of identified SLNs increasing, the portion of patients with non-SLNs metastases decreased. The number of SLNs removed was significantly correlated with non-SLNs involvement.(14–16, 19–22) The reason could be that less removed SLNs caused higher the false-negative rate. The NSABP B-32 study demonstrated that the false-negative rate was directly related to the number of removed SLNs. Thus, all nodes that qualify as SLNs should be removed, not just the bluest or hottest nodes.

Abnormal lymph nodes on imaging were significantly associated with an increased risk of non-SLNs metastases. In China, most patients did not receive ultrasound-guided fine-needle aspiration when axillary ultrasound found abnormal impalpable lymph nodes. Doctors chose to operate SLNB or directly ALND. In our study, 56.9% patients who had abnormal lymph nodes on imaging were found with non-SLN metastases. One previous study reported that it’s more likely to be preoperatively detected by axillary ultrasound if breast cancer patients had more than one ALNs metastases (23). Breast cancer patients had a higher risk of having multiple metastatic lymph nodes in a meta-analysis (24).

Previous study reported LVI (11) (16), multifocality and extranodal extension (25, 26) had association with non-SLN metastases. The present study also analyzed these factors, but the result showed that they were not associated with non-SLNs metastases (*P*>0.05). It is likely because eligible patients, number of patients, and ratio of patients with LVI, multifocality or extranodal extension in our study were not same as other studies. LVI was one of the main factors influencing the SLNs metastases, so the ratio of patients with LVI was higher than general breast cancer population.

We developed the model with pathologic invasion size, number of identified SLNs and ALN status on imaging. The performance of our model was evaluated by ROC curve with AUC=0.708. The main purpose of the present study was to assess whether omitting ALND is safe for some early-stage breast cancer patients with 1–2 sentinel lymph nodes macro-metastases and breast-conserving therapy. Our study provided useful information on the risk factors of non-SLNs metastases and showed that it still had high-risk of non-SLN metastases for a part of patients with 1–2 SLNs macro-metastases. Our model can be an auxiliary tool when doctors meet with patients with 1–2 sentinel lymph nodes macro-metastases. We can evaluate the risk of non-SLNs metastases and combine the patient’s preference, then it can result in a better treatment method.

There are several limitations to our study. Firstly, patients included in our study were from a single cancer center, which might lead to the bias of the results. Secondly, the number of patients was insufficient to do the external validation. The external validation is the best method to evaluate the predictive model. More data need to be collected in the future. In addition, the AUC was not perfect, but it could still give us some indications. The current issue needs more prospective trials to determine the safety for the patients with heavy tumor burden in ALNs.

## Conclusion

Our findings indicated pathological invasion size, number of identified SLNs and ALN status are the strongest factors influencing the non-SLNs metastases and it may not be safe of omitting ALND for the subgroup of patients with 1–2 SLNs macro-metastases and breast-conserving therapy. Our predictive model can contribute to decision-making regarding the addition of ALND and other therapy in the case of 1–2 SLNs macro-metastases and breast-conserving therapy.

## Ethical considerations

Ethical issues (Including plagiarism, informed consent, misconduct, data fabrication and/or falsification, double publication and/or submission, redundancy, etc.) have been completely observed by the authors.
